# Using genetic algorithms to optimise current and future health planning - the example of ambulance locations

**DOI:** 10.1186/1476-072X-9-4

**Published:** 2010-01-28

**Authors:** Satoshi Sasaki, Alexis J Comber, Hiroshi Suzuki, Chris Brunsdon

**Affiliations:** 1 Department of Infectious Disease Control and International Medicine, Graduate School of Medical and Dental Sciences, Niigata University, Japan; 2 Department of Geography, University of Leicester, Leicester, LE1 7RH, UK

## Abstract

**Background:**

Ambulance response time is a crucial factor in patient survival. The number of emergency cases (EMS cases) requiring an ambulance is increasing due to changes in population demographics. This is decreasing ambulance response times to the emergency scene. This paper predicts EMS cases for 5-year intervals from 2020, to 2050 by correlating current EMS cases with demographic factors at the level of the census area and predicted population changes. It then applies a modified grouping genetic algorithm to compare current and future optimal locations and numbers of ambulances. Sets of potential locations were evaluated in terms of the (current and predicted) EMS case distances to those locations.

**Results:**

Future EMS demands were predicted to increase by 2030 using the model (R^2 ^= 0.71). The optimal locations of ambulances based on future EMS cases were compared with current locations and with optimal locations modelled on current EMS case data. Optimising the location of ambulance stations locations reduced the average response times by 57 seconds. Current and predicted future EMS demand at modelled locations were calculated and compared.

**Conclusions:**

The reallocation of ambulances to optimal locations improved response times and could contribute to higher survival rates from life-threatening medical events. Modelling EMS case 'demand' over census areas allows the data to be correlated to population characteristics and optimal 'supply' locations to be identified. Comparing current and future optimal scenarios allows more nuanced planning decisions to be made. This is a generic methodology that could be used to provide evidence in support of public health planning and decision making.

## Background

Ambulance response times are a crucial factor in patient survival, especially for severe emergency cases, where the time to pre-hospital treatment is critical [1-3]. Studies have reported the association between improved survival of high risk patients with decreased response times (i.e. the time from emergency call receipt to ambulance arrival on the scene). The crucial response time is less than 4 to 5 minutes [4,5]. Additionally, ambulance response time is recognised as an important benchmark measure or indicator of pre-hospital emergency medical services (EMS) quality [6]. Other work has considered different aspects of ambulance management to improve response times, to enhance EMS management and to relocate ambulance stations [7-9]. Although statistical models for identifying the optimal location of ambulances have been discussed [10], practical methods for ambulance relocation and the evaluation of the benefits of relocation have been limited.

The need to maximise the effectiveness of public health resources is accentuated by projected population changes: the developed world is increasingly aging as longevity increases. In Japan, recent changes in the population have increased the demand for EMS and one consequence is increased ambulance response times due to reduced ambulance availability. In Niigata prefecture the volume of emergency calls has risen by 50% in 10 years from 55,434 cases in 1997 to 84,730 cases in 2007 and average ambulance response times have increased by 1.2 minutes [11]. The number of people over 65 years old in Japan has been increasing. There are two salient factors associating the elderly population to increased ambulance response times and to increased numbers of EMS case numbers. First, the aged are disproportionately high users of ambulance services [12] and they are more likely to require ambulances for life-threatening emergencies [13] such as cardiovascular and cerebrovascular disease. Second the elderly the proportion of the population that is elderly has increased and will continue to increase in the future. Currently in Japan they constitute 20% of the total population and according to future population predictions this figure will rise to up to 40% in 2050 [14]. The net effect will be to further increases EMS demands and thus response times due to ambulance unavailability.

The use of Geographic Information Systems (GIS) in the public health arena reflects a wider understanding of the importance of spatial analyses. GIS and related spatial techniques have been used as tools to analyse the spatial patterns of disease, to examine the relationship between health outcomes and accessibility, and for for better health care delivery [15-18]. There is also a role for analyses that can support public health planning, for example to maximise resource use efficiency, in the context of current and future public health needs. Genetic Algorithms (GAs) have been used extensively to develop optimal or heuristic search solutions to spatial problems in combination with GIS-based analyses. GAs are search and optimisation algorithms [19] that simulate the process of genetic mutation and selection in biological evolution. They are used when the number of possible solutions to a problem is too large to be evaluated within normal computing constraints - i.e. using deterministic methods. In the health geographics arena GAs have been compared with simulated annealing and neighbourhood search methods to determine optimal locations for hospital in Hong Kong [20]. They have been used to generate criteria weights as inputs to routing problems [21] and Genetic Algorithm for Rule-Set Production (GARP) software has been used to determine ecological niche models of monkeypox in Africa [22].

Recent work on ambulance location has included both deterministic and probabilistic models, reviewed in Schilling et al [23] and Brotocorne et al [10] respectively. Deterministic models have been used to simulate ambulance catchment areas and reduce response times based on ambulance call and dispatch logs [9], set covering models have been developed to identify ambulance facility locations accounting for the probability of vehicles being busy [24] and p-median models applied to ambulance locations in British Columbia [25]. Probabilistic models, simulating ambulance location have been developed for simple problems, for example locating 5 or 6 ambulances along a single stretch of highway [26,27] and to accommodate diurnal changes in population over a set of regular but hypothetical demand points [28]. The results are efficient computational models applied to simple real world problems, but which do not use actual EMS case data and are not evaluated in terms of improved response to EMS demands.

In this work a GA was used to model optimal ambulance locations from projected and current EMS case data. Current and predicted EMS cases were summarised over census areas and a GIS network analysis was used to measure distances between potential ambulance locations and census areas. The GA was used to model sets of potential locations for n= 2 to n= 50 ambulances from 35 current locations allowing for up to 5 ambulances at each (i.e. 175 potential locations) and evaluating them by minimising person (EMS case) distances. The GA approach is described in more detail below. The number of potential sets of locations to evaluate is large (e.g. 1.224 e+346 for 27 locations) and may be deterministically solvable. However the method needs to be able to accommodate future resource planning and needs to be able model new potential locations (i.e. not at current fire stations) along with current potential locations. Other work by the authors has identified 1255 alternative locations for fire stations and ambulances which planners may want to include in future analyses.

Network distances provide a more realistic measure of access distances and travel times than either the urban/rural characterisations for health accessibility as proxies used by Hossain and Liditka, [29] or buffer area based approaches with concentric zones around facilities as used by Mclafferty and Grady [30]. In health planning GIS-based network analyses have been extensively used to analyse service accessibility [31-33] as well as to define service catchments [32,34]. Other work has combined GIS analyses with genetic algorithms to model optimal locations in order to maximise accessibility for specific social groups [35].

The purpose of this work was to identify optimal ambulance locations based on predicted future EMS cases and to develop future EMS management strategies based on predicted demands. The modelling steps were as follows:

1. Summarise EMS case data over census areas;

2. Correlate demographic data to EMS cases;

3. Use demographic predictions to infer future EMS cases over census areas;

4. Calculate network distances between 'supply' (potential ambulance locations) and 'demand' (current and future EMS cases);

5. Model, compare and evaluate optimal current and future ambulance locations.

The research demonstrates a practical method for optimising the location of public health facilities by combining analyses of population census data with medical case data and GIS measures of access with site location optimisation using a genetic algorithm.

## Methods

### Study Area

Niigata is a prefectural capital city of 804,000 people in north western Japan. In Japan there is a one-tiered EMS system provided by the fire department [36]. Ambulances are typically located in the building of a fire station. In Niigata there are 35 fire stations which are potential locations for ambulances. Currently 27 ambulances are operated from 27 fire stations. The deployment of an ambulance to the scene is controlled by a central command system. When an emergency call is received, the nearest available ambulance to the scene is deployed to the incident.

### Data

The Niigata City Fire Bureau provided pre-hospital medical emergency records of the 21,788 cases that were attended by an ambulance in the period April to December in 2007. Of these 21,211 were geocoded and used for this study (the remainder had no locational information associated with them). From the emergency case data, the distribution of monthly ambulance users per 1,000 population were calculated for different age and gender categories. The Japanese 2005 census has 2076 census small areas in Niigata. The count of emergency cases in each census area was calculated using a point-in-polygon operation and attached as an attribute to the census data with ArcGIS v 9.2 (ESRI, USA). The centre point (centroid) was calculated for each census small area using its area (*x, y*) envelope. The road network for Niigata was provided by Increment P Corporations, Japan.

### Statistical pre-processing: calculation of future EMS cases

A prediction model to estimate future EMS demands was developed from a multivariable regression analysis. The following variables were extracted from the 2005 population census for the 2076 small areas: the counts of 0-4 years, 15-64 years, 65 years and over, and incremental age groups of every 5 years after 65 years. Additionally, counts of the number of companies employing more than five people from the enterprise census of 2008 were included in the regression. The census variables describe groups who may be more likely to need an ambulance. The employee variable describes the diurnal population flux to the city related to employment and increased numbers of people in business and industrial areas. The parameter estimates were obtained by a stepwise ordinary least squares regression analysis with SPSS ver.11 (SPSS, USA). Future EMS demands for census small areas for 5-year periods from 2015 to 2040 were estimated using projected population growth and consistent socio-economic variables using the predictive model.

The future populations of the different age groups indicated above for 2015 to 2040 were used as input of the predictive model. These were calculated from populations of 5 year age categories for males and females from the 2000 and 2005 census. The study area was divided into 38 administrative areas and the populations for both gender and each age group were calculated. The future population of the 38 administrative areas was projected using a cohort estimation method and the population estimation model developed by the National Institute of Population and Social Security, Japan [37]. A local fertility rate of 1.22 for Niigata City was used to estimate future populations. Populations in census small areas were projected in accordance with population growth rate of the 38 administrative areas in which the census small areas were located.

### Geographical analysis

Network analyses calculate the distances and travel times between locations or points on linear networks such as roads. Sets of locations are often described as 'origin and destination' or 'supply and demand'. Common applications include route finding, identifying the closest facility and the calculation of isochrones (travel time zones). In this study, census small area centroids with counts of actual and predicted cases were the 'demand' points and the ambulance stations were the 'supply' points. Network distance between the 2076 census centroids and 35 potential ambulance stations were calculated with Network Analyst (ESRI, USA). A matrix of distances from each location to each census area was generated for each time period. The distance matrices and counts of actual and predicted ambulance cases were used as input into Genetic Algorithm (GA, as described below), for the 35 potential ambulance locations using the R statistics software (The R Foundation for Statistical Computing, http://www.R-project.org).

The average time from current and GA modelled optimal ambulance locations to EMS cases were compared in order to quantify the benefit of optimising locations. An average road speed of 30 km/h was used after consultation with local ambulance service management. The EMS case records indicate whether the responding ambulance was the nearest, 2^nd ^nearest, 3^rd ^nearest, et cetera. A network analysis calculated the average times of the 27 current and 27 optimal ambulances to the individual EMS cases, taking account of which ambulance responded.

The catchment areas for each ambulance location (current and optimal) were identified from the census small areas. The network analysis identified the nearest modelled ambulance location for each census area which indicated the ambulance catchment. Catchment person distances (distance from census area centroid to ambulance location weighted by EMS cases) were used to generate measures of 'demand' for ambulance locations.

### Genetic algorithm

Genetic algorithms provide suitable models when the number of possible solutions to a problem is too large to be evaluated. For example, consider a 10 km by 10 km area with a possible location in each 100 m cell and an objective to identify the best 25 locations evaluated against some criteria. The combinations of possible solutions to evaluate even for such a small area and a small planning problem are too large to compute by brute force:

GAs create a set of potential of solutions (or 'genes') called a 'chromosome'. Pairs of chromosomes form individuals and are evaluated against some 'fitness' criteria to assess performance. If the criteria are met genes are interchanged and another generation of solutions is bred in a 'crossover'. The process of creating new individuals from successful parents continues usually for a predetermined number of cycles or until some convergence criteria are met. In order to protect against stagnation or in-breeding, mutation is introduced at regular intervals to add some random genes. In this way the GA breeds solutions by creating 'fitter' offspring and hence the analogy with natural selection: crossover creates new chromosomes from successful ones, and mutation ensures diversity. The technical details of the development of the modified grouping GA used in this work are reported elsewhere and the code may be provided on request.

In this analysis optimal current and optimal future ambulance locations were compared. The GA was used to determine sets of n= 2 to n= 50 ambulance locations from 175 potential locations using current and predicted EMS demand. The GA ran for 1500 iterations and the evaluation of fitness was based on minimising the network distance weighted by EMS case number (i.e. 'case' or person distances) from each census small area to the nearest potential ambulance location.

## Results

### Predicted EMS cases

The age and gender distribution of 21,211 emergency cases indicated that the age group of 75 years and over accounted for more than 30% of the total emergency cases (Table 1). The number of monthly users per 1,000 population among young age groups from 15 to 54 years were almost similar, whereas, the monthly users of age groups over 55 years increased incrementally. Male percentages and monthly users were greater than female.

**Table 1:** Demographic breakdown of pre-hospital emergency cases services from April to December 2007.

	Emergency cases (n = 21,211)	
	
Category	No of users (%)	Monthly users per 1,000
Age Group		
0--4	841 (4.0)	2.77
5--9	346 (1.6)	1.04
10--14	322 (1.5)	0.92
15--19	733 (3.5)	1.79
20--24	954 (4.5)	2.20
25--29	872 (4.1)	1.86
30--34	876 (4.1)	1.63
35--39	874 (4.1)	1.83
40--44	704 (3.3)	1.55
45--49	760 (3.6)	1.67
50--54	892 (4.2)	1.67
55--59	1,399 (6.6)	2.35
60-64	1,254 (5.9)	2.71
65-69	1,428 (6.7)	3.42
70-74	1,676 (7.9)	4.38
75-79	2,083 (9.8)	6.54
80-84	2,055 (9.7)	9.89
85 and over	2,585 (12.2)	13.96
Unknown	557 (2.6)	
		
Sex		
Male	10,791 (50.9)	3.05
Female	9,847 (46.4)	2.60
Unknown	573 (2.7)	

The stepwise linear regression analysis on the association between the number of emergency cases and demographic and social variables indicated that the age groups of 0 to 4 years (β = 0.006, Standard error (SE) 0.001, P < 0.001), 15 to 64 years (β = 0.021, SE 0.006, P < 0.01) and 80 years and over (β = 0.102, SE 0.006 P < 0.001), as well as the number of companies with more than five employees (β = 0.433, SE 0.013, P < 0.001), were statistically associated with number of cases. The predicted model provided a significant fit to the empirical data with a coefficient of multiple determination R^2 ^= 0.71.

**Figure 1:**
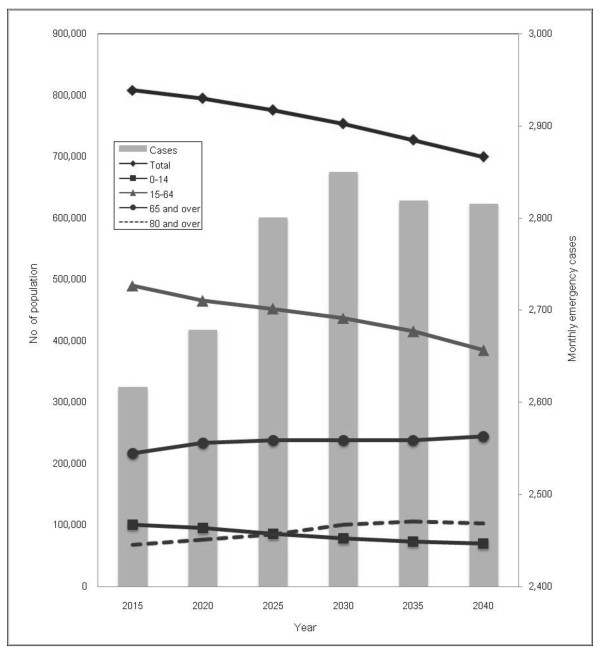
**Estimated and future demands of prehospital medical emergency services from 2010 to 2040**. Note: Emergency cases in each census small area (y) were projected with the no of population of 0-4 years (x_1_), the no of population of 15-64 years (x_2_), no of population of 80 and over (x_3_) and the no of companies with more than five employees (x_4_), based on the predicted model: y = 0.006*x_1_+0.021*x_2_+0.102*x_3_+0.433*x_4_-0.268.

The projected populations from 2015 to 2040 showed that total population would decrease from approximately 814,000 in 2005 to 699,000 in 2040 (Figure 1). The population of age groups of '0-4 years' and '15-64 years' were estimated to decrease, whereas the projected population of age groups of '65 and over', and '80 and over' as a significant predictor of EMS demands were predicted to increase. Accordingly, the number of monthly emergency cases in the future indicated a 20% increase from 2007 to a peak in 2030 and after which period the number of emergency cases was estimated to decrease to 2040.

### Spatial analyses using the genetic algorithm

**Figure 2:**
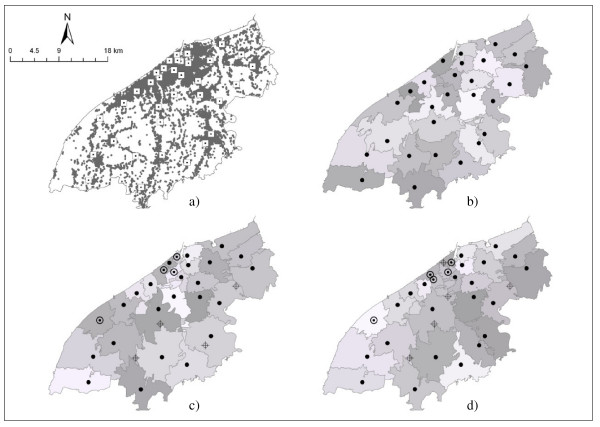
**Emergency cases and current and optimal ambulance locations with their catchment areas**. a) Distribution of emergency cases and 35 fire stations (potential ambulance locations). b) Current location of 27 ambulances. c) The optimal location of 27 ambulance stations based on emergency cases in 2007. d) The optimal location of 27 ambulance stations based on predicted emergency cases for 2030. The following legend applies to figures 3c) and 3d): solid circle - optimal locations that are the same as current locations; ringed circle - new locations; hollow circle with a cross - current sites not selected during optimisation.

The geocoded 21,111 emergency records and the 35 potential ambulance locations are shown in Figure 2a. Current emergency cases are clustered in centre of the city where fire stations were also concentrated. The current distribution of 27 ambulances is shown in Figure 2b. The GA was used to identify alternative sets of ambulance locations from the 35 potential sites based on current EMS cases. The results in Figure 2c indicate that current ambulance locations could be improved to better serve current needs by re-allocating resources. The results of the GA suggest that four ambulances out of 27, three in the urban north and one in the south west, could be reallocated from the more rural areas in the south of the city in (Figure 2b, 2c). The analysis of future optimal locations was based on predicted cases for 2030, when EMS demands were predicted to reach a peak. There are different patterns of resource allocation in the more urban areas (which are northerly and with smaller catchments) compared to optimal location based on current emergency cases and to the current ambulance distribution. Comparison with 2007 indicates that some of those ambulance locations in peri-urban areas, rejected in 2007, may be needed in 2030 (Figure 2d).

The benefit of optimising ambulance location was quantified in terms of travel time using a network analysis. Travel times cases from the set of optimised locations to current emergency cases were compared with those from current ambulance locations. Average times were reduced by 57 seconds from the current ambulance locations to the optimised ambulance locations (5 minutes and 21 seconds and 4 minutes and 24 seconds respectively). Of the actual EMS cases, 67.8% were attended by their nearest ambulance, whereas when optimally located, simulation analysis indicated that 83.0% of the cases were be covered by their nearest ambulance.

**Figure 3:**
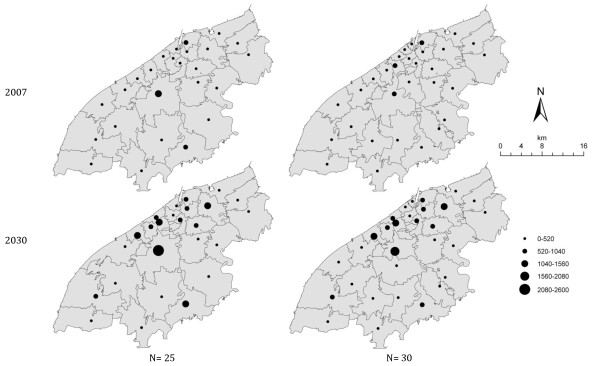
**A comparison of the optimised location for *n = 25 *(left) and *n = 30 *(right) ambulances**. These are evaluated against current emergency cases in 2007 (top) and predicted emergency cases in 2030 (bottom). The size of the point indicates the 'demand' in terms of emergency case distances.

The ambulance locations identified by the GA were evaluated to highlight which were under- or over-used based on the number of actual and predicted cases and their distances to them. The total person distances in each ambulance catchment were calculated from the number of cases in each census area and the distance from census area centroid to ambulance location. This provides an index of ambulance station 'busyness' and accounts for the number of cases and the distances. By way of example Figure 3 compares the demand and location of n = 25 and n = 30 ambulances in 2007 and in 2030 (these numbers were chosen to illustrate the differences around the current 27 ambulances). The increased demand is evident in 2030 in both cases and changing spatial distribution of demand could be used to prioritise additional ambulance facilities at specific locations. Figure 3 shows a pattern of increased ambulance demand (describing distances and cases) in the peri-urban areas for 2030 compared to the current distribution of demand. In some instances the GA has suggested ambulance locations, where the catchment does not include large person distances, but where an ambulance is still needed. In others, locations are close together but still expected to be busy.

## Discussion

Reallocation of ambulances to optimal locations has the potential to improve service delivery. Response time is a crucial determinant to survival of life-threatening emergency events. Studies on the effect of the reducing ambulance response times on survival rates have argued that mortality risk is sensitive to response times of less than 5 minutes [4]. Additional survival benefit is not associated with improved response times beyond this figure. Therefore, allocation of ambulances able to reach potential emergency cases in their catchment areas within 5 minutes is of great importance. In our study the optimal locating ambulances reduced the estimated average response times from 5 minutes 21 seconds to 4 minutes 24 seconds and increased the percentages of the deployment of ambulances nearest to the scene from 67.8% to 83.0%. Allocating ambulances to areas of high EMS need reduces the possibility more distant ambulances being deployed. The deployment percentages of the nearest ambulance and reduced response times indicate more efficient ambulance utilisation.

In this analysis, potential ambulance locations were identified by minimizing the person-distance to the ambulance station. Determining ambulance 'demand' at different times provides evidence to support planning and allows optimal location strategies at different times to be compared. Future service demand was modelled by relating current cases to demographic data and applying that relationship to predicted population changes. We note that the current optimal locations may be different from future optimal locations. Providing planners with a time series of EMS (or other service) demands based on predictive models that indicate the service resources that are required, allows holistic analyses of the costs and benefits of any re-allocation strategy over time.

The approach adopted by this research summarised EMS case data over census areas. Using census areas allowed EMS data to be correlated with demographic variables, which in turn enabled future emergency cases to be predicted and facilitated a GIS-based network analysis. The application of a genetic algorithm to determine network distances between service 'supply' and patient 'demand' points allowed optimal service locations to be modelled. In this case the evaluation criterion was to minimise 'case distances' and the benefits of service relocation were quantified in terms of reduced ambulance response times. We note that i) the results provide evidence to support planning decisions, ii) the method can be adjusted to accommodate different sets or types of service locations, iii) census areas provide a convenient spatial unit over which to summarise other data, and iv) different sets of evaluation criteria could be selected according to local priorities. These include the trade-offs between the benefits of optimal location and the associated costs of facility relocation.

The level of severity of emergency cases is a critical factor to be considered for improving survival rates. There is a survival benefit from minimising response time for patients with intermediate or high risk of mortality [5]. Screening EMS cases for the level of severity (triage) in order to deploy rapid and intensive ambulances to life-threatening medical events will further reduce inefficient ambulance utilization. Studies on the appropriateness of ambulance transportation suggested that between 28% and 44% of ambulance transportation were considered as unnecessary emergency services [38,39]. In the United States, field triage systems and criteria for screening emergency events have been established and several evaluation studies have been conducted for further development [40-42]. In some countries, EMS systems have developed posting strategies that position ambulance to be in close proximity to emergency calls within high demand service areas during peak hours [4]. In this study severity categorisation of the cases was not applied, however, further analysis on allocation of optimal ambulance for different level of severity has been undertaken. Further work will also consider the temporal fluxes involved in ambulance demand.

Monitoring population trends (age groups) are of importance when estimating EMS demands as those demands will increase in the future as the population ages. Our study supports the view that, for the population beyond middle age, the utilization of EMS increases exponentially with age [13,14,43]. The population of over 80 years is a significant predictor of EMS demands compared to other age groups due to increased risk of life-threatening disease such as cardiovascular and cerebrovascular disease. Analysis at the census area level allows the impacts of predicted population changes to be evaluated.

We note that this study has several limitations and that improving some of the data aspects may improve the modelled outputs. The EMS data and census data are not concurrent which is necessarily the case for nearly all studies that involve the use of census data. The assumption when using census has to be that the population fluxes are not significantly large in comparison to the other phenomena for which data have been collected (e.g. EMS cases). The emergency case data was for a period of 9 months and may not be representative. However, since seasonal difference of geographic distribution for the period was limited, we assume that the results of the optimal location are valid to emergency events. The number of companies with more than five employees was identified as a significant positive determinant for EMS demand prediction. We used data from the national enterprises census of 2008, and that may result in overestimation of EMS future demands since overall population was estimated to decrease. Further work is required to estimate EMS future demands with consideration of social, business and city planning indicators. It is possible to identify a number of other potential limitations: the road network does not account for the traffic density patterns in space and time; road network distances were used to estimate improved EMS response as a result of re-allocation; and future EMS demands were based on linear projections. We can present arguments for these choices based on pragmatism: it was beyond the scope of this project to capture data on traffic density; we sought to make a like for like comparison of travel times; we have no indication that the relationships amongst the variables correlated with EMS are not linear. Such criticisms could be used to construct an argument for improved data collection in which case the method and results we present in this work demonstrate a proof of concept, although the sensitivity of the results indicate consistent trends regardless of the number of ambulances and the underpinning current and projected EMS cases.

Measuring and predicting physical access to services such as public health is important for service delivery and for planning future requirements. In this work we found census areas to provide a convenient unit over which to summarise point data and to integrate other data (socio-economic, demographic, etc). Network analysis using census area centroids to analyse access to facilities extends this functionality. Statistical and spatial analyses can then be used to determine how well different social groups are served by those facilities [44] and how those facilities could be re-arranged to better serve targeted social groups [35]. The combination of standard techniques that incorporate demographic data, GIS network analysis and spatial statistical techniques have been shown to answer complex problems in a number of planning domains.

## Conclusions

Re-allocation of ambulances contributes to improved response times to the scene. Since response time is critical factor to survival rates of life-threatening emergency cases, reducing the response time has the potential to improve patient survival. The methods used in this work can be applied to quantify and optimise location, number and physical access for any type of health facilities and for any target demographic group. Such evaluations are increasingly important for planning future health service requirements and locations, as well to demonstrate the equity of service being delivered especially in the public sector.

## Competing interests

The authors declare that they have no competing interests.

## Authors' contributions

SS compiled and analysed the EMS case data to generate future EMS cases. AJC applied and ran the GAs, interpreted the outputs and drafted the manuscript. HS participated in the design of the study and commented on the manuscript. CB developed the genetic algorithm and provided guidance on its application. All authors read and approved the final draft.
